# Natural strategies to optimize estrogen levels in aging women: mini review

**DOI:** 10.3389/fragi.2025.1706117

**Published:** 2025-11-25

**Authors:** Olena Bolgova, Inna Shypilova, Volodymyr Mavrych

**Affiliations:** 1 College of Medicine, Alfaisal University, Riyadh, Saudi Arabia; 2 School of Medicine, St. Matthew’s University, George Town, Cayman Islands

**Keywords:** estrogens, phytoestrogens, micronutrients, gastrointestinal microbiome, postmenopause, life style, dietary supplements

## Abstract

**Introduction:**

Menopause triggers declining estradiol, causing vasomotor symptoms, bone loss, and urogenital changes. Despite hormone therapy’s effectiveness, safety concerns drive 40%–50% of Western women toward natural alternatives.

**Aim:**

To evaluate evidence supporting non-pharmacological interventions that modulate endogenous estrogen activity in postmenopausal women.

**Methods:**

48 high-quality publications (2015–2025) examining dietary interventions, micronutrient supplementation, gut microbiome modulation, lifestyle modifications, and botanical remedies for menopausal symptoms were analyzed and included in this review. Selection criteria included randomized controlled trials, systematic reviews, meta-analyses, and cohort studies specifically addressing natural interventions in perimenopausal and postmenopausal women.

**Results:**

Multiple natural approaches demonstrated clinically meaningful effects. Dietary phytoestrogens (50–80 mg/day isoflavones) reduced severe hot flashes by up to 92%, improved metabolic parameters, and were confirmed safe for reproductive tissues. Flaxseed lignans reduced perimenopausal symptoms. Combined vitamin E and omega-3 lowered hot flush intensity, while vitamin E alone showed estrogenic receptor activation. Curcumin (500 mg/day) reduced hot flashes after 4 weeks and improved metabolic profiles. Probiotics containing L. brevis KABP052 increased circulating estrogens by up to 26% over 12 weeks. Stress reduction interventions improved quality of life, and cognitive behavioral therapy reduced insomnia severity. Botanicals including black cohosh, red clover, and rhapontic rhubarb reduced vasomotor symptoms, while resveratrol (75 mg twice daily) significantly improved bone mineral density over 12 months. Research gaps remain regarding dosing and genetic variability.

**Conclusion:**

A multi-domain approach incorporating phytoestrogen-rich foods, targeted micronutrients, gut microbiome optimization, and regular exercise provides evidence-based options for managing estrogen decline. While these approaches cannot fully replace hormone therapy, they provide valuable alternatives for women with contraindications or preferences against pharmaceutical intervention. Future research should focus on personalized approaches incorporating genetic profiling.

## 1 Introduction

Estrogen deficiency after menopause accelerates chronic disease risk and diminishes quality of life (Hulteen et al., 2023). Menopause occurs at a mean age of 48.8 years, with remarkably little geographic variation, and estrogen decline drives vasomotor symptoms, genitourinary changes, sleep disturbances, and cognitive alterations (Davis and Baber, 2022). Untreated estrogen deficiency significantly increases risks of cardiovascular disease, accelerated bone mineral density loss leading to osteoporosis, and cognitive decline. Epidemiological data demonstrate that women experiencing severe vasomotor symptoms have a 1.5-fold increased risk of coronary heart disease and stroke compared to asymptomatic women (Zhu et al., 2020). The North American Menopause Society (NAMS) and International Menopause Society (IMS) guidelines recommend menopausal hormone therapy as the most effective first-line treatment for moderate-to-severe vasomotor symptoms in eligible women, with non-hormonal prescription medications such as SNRIs, gabapentin, or clonidine as second-line options for women with contraindications to estrogen (The 2022 Hormone Therapy Position Statement of The North American Menopause Society Advisory Panel, 2022). Even though menopausal hormone therapy (MHT) can effectively mitigate these symptoms (Newson, 2016), among those women who were opposed to MHT, 25% indicated that they were afraid of the increased risk of breast cancer, 34% cited cardiovascular risks, and 26% were worried about weight gain (Depypere et al., 2016). Recent concerns about MHT risks have prompted 40%–50% of Western women to seek complementary approaches (Franco et al., 2016).

Dietary supplements are a global business worth more than US$100 billion annually (Binns et al., 2018). The imperative, therefore, is to critically appraise which natural strategies are biologically plausible, safe, and supported by contemporary human evidence.

The natural interventions reviewed in this paper operate through multiple physiological mechanisms: phytoestrogens act as selective estrogen receptor modulators with varying tissue affinities; micronutrients support enzymatic pathways critical for estrogen synthesis and metabolism; gut microbiota modulation enhances estrogen reabsorption through β-glucuronidase activity; and lifestyle interventions optimize neuroendocrine signaling pathways affected by estrogen decline.

This comprehensive review examines current evidence on natural strategies to optimize estrogen levels in aging women, focusing on recent advances, controversies, and future directions.

## 2 Dietary phytoestrogens: mechanisms and clinical evidence

### 2.1 Soy isoflavones

Soybeans contain genistein and daidzein, which act as selective estrogen receptor modulators (SERMs) with preferential β-receptor binding (Messina, 2016; Křížová et al., 2019). In the 12-week randomized study, a low-fat vegan diet supplemented with soybeans significantly increased isoflavone intake, reduced mean body weight, and decreased severe hot flashes by 92% compared with controls. Multivariable analyses identified increased daidzein intake, rather than weight loss, as the primary independent predictor of hot flash reduction (Kahleova et al., 2025). In another study, retrospective data from 200 menopausal women showed that daily supplementation with myo-Inositol, cocoa polyphenols, and soy isoflavones (80 mg, including 50 mg genistin) in menopausal women with metabolic syndrome was associated with significant improvements in vasomotor symptoms, particularly reducing the frequency and severity of hot flushes over 6 months. These findings support the beneficial role of soy isoflavones as part of a combined natural approach for managing menopausal symptoms (Mainini et al., 2024).

Equol, an active metabolite possessing estrogen-like activity, is produced by the action of intestinal microbiota on soy isoflavones. The 2017 cross-sectional study demonstrated that equol producer status was associated with favorable metabolic parameters in women in the early phase of postmenopause, with transitional periods noted during the decline in intrinsic estrogen levels (Yoshikata et al., 2017).

Meta-analyses from the past decade have strengthened evidence supporting phytoestrogen efficacy for specific menopausal symptoms. Chen et al. (2015) found that phytoestrogens significantly reduced hot flush frequency compared to the placebo, while Franco et al. (2016) reported modest reductions in hot flash frequency and vaginal dryness, although without significant effects on night sweats.

A 2022 meta-analysis by Boutas et al. found a clear inverse correlation between isoflavone consumption and breast cancer risk in both pre- and postmenopausal women, addressing longstanding safety concerns (Boutas et al., 2022). The natural interventions reviewed in this paper operate through multiple physiological mechanisms: phytoestrogens act as selective estrogen receptor modulators with varying tissue affinities; micronutrients support enzymatic pathways critical for estrogen synthesis and metabolism; gut microbiota modulation enhances estrogen reabsorption through β-glucuronidase activity; and lifestyle interventions optimize neuroendocrine signaling pathways affected by estrogen decline. The European Food Safety Authority recently concluded that isoflavones do not adversely affect breast, thyroid, or uterine tissue in postmenopausal women (Messina, 2016).

### 2.2 Flaxseed lignans

Secoisolariciresinol diglucoside (SDG) is metabolized to enterodiol and enterolactone, exerting anti-oxidant, anti-cancerous, anti-inflammatory, modulation of gene expression, anti-diabetic, estrogenic, and anti-aromatase effects (Plaha et al., 2022). A recent randomized trial indicated that flaxseed supplementation significantly reduced the severity of perimenopausal symptoms (hot flushes, heart discomfort, sleep problems, depressive mood, irritability, anxiety, dryness of vagina, *etc.*) compared with placebo (Shrivastava et al., 2025).

### 2.3 Other dietary sources

Legumes (such as lentils and chickpeas), sesame, and dried fruits provide additional lignans and coumestans, two distinct classes of phytoestrogens (plant compounds with estrogen-like activity broadly grouped with isoflavones) (Desmawati and Sulastri, 2019). While not specific to menopausal women, the metabolic benefits of phytoestrogen-rich foods are particularly relevant to this population, as postmenopausal women face increased metabolic syndrome risk due to estrogen decline. A 2017 study of 5,426 participants recommends higher consumption of fruits, coarse cereals, and soy products, which was associated with a lower prevalence of metabolic syndrome in the suburban population of Shanghai (Wei et al., 2023). This finding supports the broader cardiovascular and metabolic protective effects of phytoestrogen-rich dietary patterns, which become increasingly important during the menopausal transition when metabolic dysfunction accelerates.

## 3 Lipid-soluble micronutrients in estrogen metabolism

### 3.1 Vitamin E and omega-3 fatty acids

A recent meta-analysis of 10 studies (1,100 participants) found that combined vitamin E and omega-3 reduced hot flush intensity, while either alone had no significant effect (Maghalian et al., 2022). Additionally, vaginal vitamin E has been studied for atrophic vaginitis, showing improvements in symptoms, vaginal pH, and maturation index (Feduniw et al., 2022). Vitamin E compounds have been shown to exhibit estrogenic activity, activating ERα/ERβ and stimulating ER-dependent gene transcription (Khallouki et al., 2016).

### 3.2 Curcumin

A 6-month intervention in healthy postmenopausal women observed within-group increases in serum estradiol levels, improved the lipid profiles and serum glycemic indices in participants receiving nanomicelle curcumin capsules (Sadeghzadeh et al., 2023). Oral curcumin (500 mg/day) for 8 weeks significantly reduced the number of hot flashes in postmenopausal women, with the first significant improvement observed after 4 weeks. Curcumin had no significant effect on anxiety, sexual function, or other menopausal symptoms (Ataei-Almanghadim et al., 2020).

## 4 Gut microbiome and the “estrobolome”

The emerging concept of the “estrobolome” represents one of the most significant recent developments in understanding how the microbiome influences estrogen metabolism. New research suggests that specific bacterial strains may be targeted to optimize estrogen reabsorption, potentially offering a novel approach to managing menopausal symptoms without the need for direct hormone supplementation.

The gut microbial gene repertoire (estrobolome) regulates enterohepatic estrogen recycling through β-glucuronidase activity (Kumari et al., 2024). Menopause-related dysbiosis (lack of *Lactobacillus* and elevated levels of *Clostridia*) decreases reabsorption of conjugated estrogens. When this process is impaired through dysbiosis, characterized by lower microbial diversity, circulating estrogens are reduced (Baker et al., 2017).

A 12-week randomized, placebo-controlled trial showed that a probiotic containing L. brevis KABP052 maintained serum estrogen levels in peri- and postmenopausal women, while placebo recipients experienced declines. After 12 weeks, estradiol (31.6 vs. 25.1 pg/mL) and estrone (21.4 vs. 13.2 pg/mL) were higher in the probiotic group. This effect is likely mediated by gut microbial β-glucuronidase, which reactivates estrogen glucuronides for reabsorption (Honda et al., 2024).

A 2013–2016 study with 2,699 participants indicated that probiotic consumption was associated with higher estradiol levels in premenopausal women and lower testosterone levels in postmenopausal women. Probiotic intake may represent a supportive strategy for managing hormonal transitions and their associated symptoms throughout a woman’s life cycle (Zou et al., 2023).

## 5 Lifestyle interventions: exercise, stress reduction, and sleep regulation

### 5.1 Exercise

A meta-analysis of 18 randomized controlled trials (approximately 2,000 participants) found that physical activity significantly reduces total and free estradiol, with stronger effects in non-obese women and with high-intensity exercise (Ennour-Idrissi et al., 2015), subgroup analyses indicated that this effect is independent of menopausal status and is more noticeable in non-obese women and those who perform high-intensity exercise. Another meta-analysis of 40 studies (2,230 postmenopausal women) found that all exercise modalities significantly improved bone mineral density at the lumbar spine, total hip, femoral neck, trochanter, and total body. Combined aerobic and resistance training was particularly effective, highlighting regular exercise as a non-pharmacological strategy for mitigating bone loss and supporting skeletal health in postmenopausal women (Hejazi et al., 2025).

Recent research by Marsh et al. demonstrated that exercise has adipocyte-specific effects that directly counteract the negative metabolic impact of estrogen loss (Marsh et al., 2023). Geraci et al. found that exercise stimulates satellite cell proliferation in skeletal muscle, partially compensating for estrogen-related muscle dysfunction during menopause (Geraci et al., 2021). These findings demonstrate that exercise provides critical benefits through estrogen-independent mechanisms, making it an essential component of menopausal health management despite modest reductions in circulating estradiol.

The safety profile of lifestyle interventions is excellent, with minimal risk of adverse effects when exercise is tailored to individual fitness levels and pre-existing conditions. Stress reduction and sleep hygiene interventions show no significant adverse effects in clinical trials (Hejazi et al., 2025).

### 5.2 Stress reduction

The meta-analysis of five randomized controlled trials (475 participants) indicated that the mindfulness-based intervention groups showed significant improvements in total quality of life and vasomotor and physical quality of life, compared to control groups (Chen et al., 2021).

### 5.3 Sleep regulation

Insomnia occurs in almost 60% of postmenopausal women (Hachul et al., 2023). Sleep fragmentation elevates evening cortisol, suppressing hypothalamic GnRH and downstream estradiol (AvivaCohn et al., 2023). Morssinkhof et al. found that higher endogenous estrogen correlates with better sleep quality, providing a physiological explanation for menopausal sleep disruption (Morssinkhof et al., 2020).

Up-to-date developments in digital sleep tracking and home-based Cognitive behavioral therapy for insomnia (CBT-I) applications have made sleep interventions more accessible, representing an emerging area where technology may enhance the management of menopausal symptoms through improved sleep regulation (Guthrie et al., 2018).

## 6 Botanical medicines and standardized extracts

### 6.1 Black cohosh (*Cimicifuga racemosa*)

In a retrospective cohort study of 174 symptomatic menopausal women, *Cimicifuga racemosa* extract and MHT were compared. Both therapies significantly improved Menopause rating scale scores, while neither treatment affected body weight nor metabolic parameters (Friederichsen et al., 2020). Recent research has clarified that black cohosh operates primarily through serotonergic mechanisms rather than direct estrogenic activity (Mohapatra et al., 2022).

Black cohosh is widely used for relief of menopausal symptoms, but evidence for its efficacy remains limited. Adverse effects, including rare severe hepatotoxicity, and frequent product adulteration (found in 42%–67% of samples), raise safety concerns. More standardized, well-controlled studies are needed to clarify its long-term safety and effectiveness (Le et al., 2025).

### 6.2 Red clover (Trifolium pratense)

A meta-analysis of eight trials assessing red clover isoflavones for hot flashes and menopausal symptoms in peri- and postmenopausal women indicated that this treatment significantly reduced the daily number of hot flashes, with the greatest effects observed in postmenopausal women experiencing more than five hot flashes per day (Kanadys et al., 2021).

### 6.3 Rhapontic rhubarb (*Rheum rhaponticum*)

A systematic review and meta-analysis of four studies (encompassing 390 participants) found that supplementation with ERr 731®, a standardized Rheum rhaponticum root extract, significantly reduced Menopause Rating Scale scores compared to the control (Dubey et al., 2024).

### 6.4 Other emerging botanicals

Dong quai, chasteberry, and Viburnum opulus show preliminary benefit, but data are limited to small, heterogeneous trials (Dietz et al., 2016). Kiyama identified ginger compounds with estrogenic activity that may improve menopausal symptoms beyond their traditional use for nausea (Kiyama, 2020).

### 6.5 Resveratrol: breakthrough in bone health

Wong et al. demonstrated that regular resveratrol supplementation (75 mg twice daily) over 12 months improved bone mineral density in the lumbar spine and femoral neck of postmenopausal women. Benefits were greater in participants with poor baseline bone health markers, and improvements in femoral neck T-score correlated with enhanced perfusion (Wong et al., 2020).

The identification of resveratrol’s specific effects on bone health represents one of the most significant recent breakthroughs in non-hormonal approaches to managing the risk of postmenopausal osteoporosis. This emerging evidence offers a promising avenue for women seeking alternatives to bisphosphonates and other conventional bone-preserving medications.

## 7 Discussion

Recent evidence supports specific natural approaches for managing estrogen decline in aging women, particularly phytoestrogens for vasomotor symptoms and bone health, black cohosh for hot flashes, and exercise for metabolic protection. However, significant methodological limitations and inconsistencies persist across studies. The evidence for effective treatment of postmenopausal symptoms using natural approaches continues to evolve, highlighting the need for well-designed, large studies with standardized endpoints.

### 7.1 Different schools of thought

The field of menopausal symptom management through natural approaches reveals several divergent perspectives. Traditionally, some clinicians have advocated for a “hormonal deficiency” model, viewing menopause primarily as an estrogen deficiency state requiring hormone replacement. This model contrasts with the emerging “adaptive transition” perspective, which frames menopause as a natural life stage requiring supportive rather than replacement strategies.

The “functional medicine” approach emphasizes personalized interventions based on individual hormone metabolism patterns, advocating for comprehensive testing and targeted natural interventions. In contrast, the “evidence-based integrative” perspective prioritizes interventions with the strongest clinical trial support, regardless of mechanistic explanations.

Regional variations in approaches are notable. Asian medical traditions have long incorporated soy foods and herbal remedies into menopausal care, whereas European approaches more commonly feature standardized botanical extracts, such as black cohosh and rhapontic rhubarb. North American perspectives often emphasize lifestyle modifications and pharmaceutical-grade supplements.

### 7.2 Controversies and safety considerations

The safety profile of phytoestrogens in estrogen-sensitive conditions remains contentious. Present evidence demonstrated that taking in dietary isoflavone helps reduce the breast cancer risk (Yang et al., 2023); however, caution is still advised in estrogen-receptor-positive breast cancer survivors. Botanicals with unstandardized phytoestrogen content pose theoretical proliferative risks that require further investigation (Ziaei et al., 2017).

Herb-drug interactions represent another safety concern. Black cohosh demonstrates weak CYP3A4 inhibition (Sprouse and van Breemen, 2016), while red clover may potentiate warfarin’s anticoagulant effects (Karimpour-Reihan et al., 2018). The appropriate dosing and standardization of botanical products remain inconsistent, hindering the development of reliable clinical recommendations and raising questions about product quality (Mohapatra et al., 2022).

Genitourinary symptoms present a particular challenge. A systematic review by Trinchieri et al. found that vitamin D, phytoestrogens, and estrogen modulators showed inconsistent results for genitourinary symptoms (Bapir et al., 2023). Treatment strategies for postmenopausal urinary incontinence vary widely, but comparison is difficult due to heterogeneous protocols and outcome measures.

### 7.3 Current knowledge gaps

Recent advancements in understanding estrogen metabolism have illuminated several critical research gaps. Most clinical trials examining natural approaches have been limited to durations of under 12 months, leaving the long-term effects on bone, cardiovascular, and cognitive outcomes largely unknown. Additionally, studies have predominantly focused on Caucasian women under 70 years, with limited representation of diverse populations or the oldest age groups. The influence of genetic polymorphisms affecting estrogen metabolism (CYP19, COMT, ESR1) remains poorly understood, despite their potential to modify individual responses to interventions. Interaction effects between multiple natural approaches are rarely investigated, which limits our understanding of the synergistic benefits. Furthermore, personalized approaches based on genetic profiles, microbiome composition, and symptom clusters require substantial development.

Interaction effects between multiple natural approaches remain poorly understood, limiting guidance on optimal combination strategies. Research on personalized approaches based on genetic profiles, microbiome composition, and symptom clusters is still in its infancy, though it represents one of the most promising directions for future investigation.

### 7.4 Potential future developments

Future research directions should emphasize the integration of multi-omics to predict phytoestrogen responsiveness and develop personalized medicine approaches that incorporate genetic and microbiome testing. The development of standardized, quality-controlled botanical formulations with verifiable bioactive content will enhance clinical reliability and accuracy. Early intervention strategies during perimenopause may offer preventative benefits before significant symptom burden develops. The integration of digital health technologies for symptom tracking and intervention optimization represents an emerging frontier, as do hybrid e-health trials utilizing wearable sensors for real-time vasomotor monitoring. Ultimately, the development of botanical reference standards and regulatory frameworks comparable to those for pharmaceuticals would significantly enhance product reliability and clinical confidence.

The optimal approach to managing menopausal estrogen decline involves individualized, integrative strategies combining dietary phytoestrogens, targeted micronutrients, gut microbiome optimization, and structured exercise with sleep hygiene interventions. These combined approaches may raise circulating estradiol while attenuating menopausal symptoms when tailored to individual needs.

## 8 Conclusions

Natural approaches to menopausal estrogen decline offer evidence-based alternatives for women unable or unwilling to use hormone therapy. A multi-domain approach incorporating phytoestrogen-rich foods (working through selective estrogen receptor modulation), targeted micronutrients (supporting estrogen cofactors), gut microbiome optimization (enhancing enterohepatic estrogen recycling), and regular exercise (improving metabolic signaling independent of estrogen) provides evidence-based options for managing estrogen decline. While these approaches cannot fully replace hormone therapy, they provide valuable alternatives for women with contraindications or preferences against pharmaceutical intervention. Future research should focus on personalized approaches incorporating genetic profiling.
